# Development and Validation of a RPLC Method for the Determination of 2-Phenoxyethanol in Senselle Lubricant Formulation

**DOI:** 10.4103/0250-474X.70476

**Published:** 2010

**Authors:** G. A. Shabir, T. K. Bradshaw, G. Q. Shar, S. A. Arain

**Affiliations:** Oxford Brookes University, School of Life Sciences, Headington Campus, Oxford, UK; 1 University of Sunderland, Sunderland Pharmacy School, Sunderland, UK; 2 University of Strathclyde, Department of Pure and Applied Chemistry, Glasgow, UK

**Keywords:** HPLC, method validation, senselle lubricant formulation, 2-phenoxyethanol

## Abstract

A new and simple reversed-phase liquid chromatographic method has been developed and validated for the determination of 2-phenoxyethanol preservative (0.3%, w/w) in senselle lubricant formulation. The separation was achieved with acetonitrile-tetrahydrofuran-water (21:13:66, v/v/v) as mobile phase, a C_8_ column, and UV detection at 258 nm. The calibration curve is linear (r^2^= 0.9999) from 20-140% of the analytical concentration of 0.75 mg/ml. The mean percent relative standard deviation values for intra- and inter-day precision studies are <1%. The recovery of 2-phenoxyethanol ranged between 99.76 and 100.03% from lubricant formulation. The limits of detection and quantitation are determined to be 0.094 and 0.15 mg/ml, respectively. The method was found to be robust and can be successfully and reliably used to determine the 2-phenoxyethanol preservative content of marketed formulations.

2-Phenoxyethanol (ethylene glycol monophenyl ether, C_8_H_10_O_2_, fig. 1) has been widely used as preservative in cosmetics, skin care products, toiletry, sexual lubricant products and pharmaceutical applications (i.e. in vaccine formulations)[1,2], because of its broad antimicrobial spectrum with good stability and non-volatility[3]. It is a good general bactericide (most active against gram negative bacteria) but a weak fungicide and is generally used in combination with other preservatives. Some liquid chromatographic (LC) methods have been reported for the determination of 2-phenoxyethanol with combination of other components[4,5]. These reported methods are complicated and time consuming with poor chromatographic separation and longer analytical run time. Furthermore, forced degradation decomposition studies were not included in this work. Determination of 2-phenoxyethanol with solid phase microextraction-gas chromatography-mass spectrometry (SPME-GC-MS/MS) detection has also been reported[6], but this technique is not common use in pharmaceutical quality control laboratories. LC technique (fig. 2) has been widely used in pharmaceutical analysis in quality control laboratories because of its sensitivity and specificity. The purpose of this study was to develop and validate a rapid, cost-effective and selective reversed-phase liquid chromatographic (RPLC) method for routine quality control analysis of 2-phenoxyethanol. Stress testing of the preservative was also conducted, as required by the International Conference on Harmonization (ICH)[7] to support the suitability of the method. As a best practice[8–12] in the subsequent investigation, the new RPLC method was validated according to ICH and U.S. Food and Drug Administration (FDA)[13–16] guidelines.

**Fig. 1:**
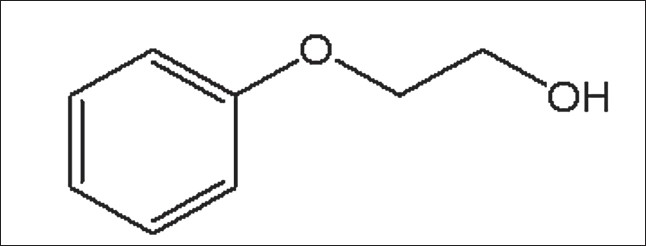
Chemical structure of 2-phenoxyethanol

**Fig. 2:**
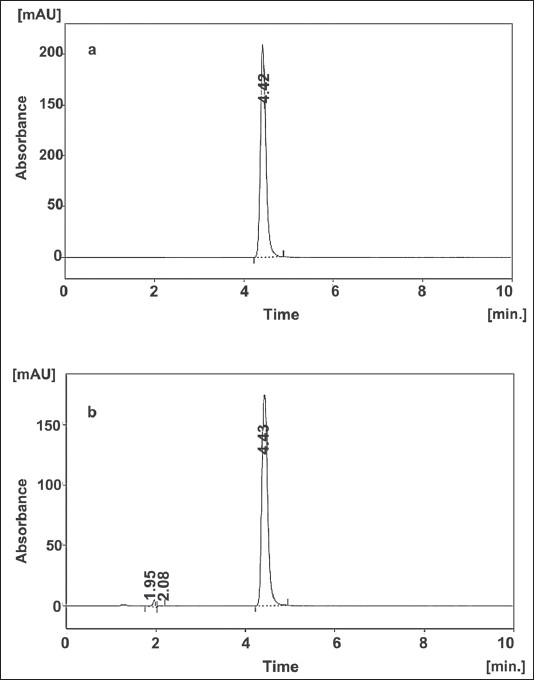
LC chromatogram of standard 2-phenoxyethanol and sample LC chromatogram of 2-phenoxyethanol, (a) standard reference; (b) chromatogram of sample showing well resolved peak of 2-phenoxyethanol at retention time 4.43 min from the impurity peaks at retention time 1.95 and 2.08 min.

## MATERIALS AND METHODS

Methanol (HPLC-grade), tetrahydrofuran (HPLC-grade), 2-phenoxyethanol (pure >99%), and formic acid were purchased from Sigma-Aldrich (Gillingham, UK). Distilled water was de-ionised by using a Milli-Q system (Millipore, Bedford, MA). A Knauer HPLC system (Berlin, Germany) equipped with a module 1000 LC pump, 3950 autosampler and 2600 photodiode-array (PDA) detector was used. The data were acquired via ClarityChrom data acquisition software. Separation was achieved using Lichrosorb C_8_ column (150×4.6 mm i.d., 5 mm particle size) from Jones Chromatography (Hengoed, UK). All chromatographic experiments were performed in isocratic mode. The mobile phase consisted of a mixture of acetonitrile-tetrahydrofuran-water (21:13:66, v/v/v) pH 3.0. The pH was adjusted with formic acid. The flow rate was 1 ml/min, the injection volume was 10 ml, and the temperature was set at 35°. Chromatograms were recorded at 258 nm using UV detector.

### Standard and sample preparation:

2-Phenoxyethanol standard solutions at 0.75 mg/ml were prepared by dissolving approximately 75 mg of 2-phenoxyethanol in 100 ml mobile phase. An accurately weighed amount (1.25 g) of 2-phenoxyethanol sample lubricant was dissolved in 50 ml mobile phase, yielding a final concentration of 25 mg/ml. The sample was filtered through a sample filtration unit (0.45 μm) and injected into the LC system.

### Method validation:

The method was validated for linearity, range, precision (intermediate precision and repeatability), accuracy, specificity, limits of detection and quantitation. Calibration standard solutions of 2-phenoxyethanol were prepared at concentrations of 0.15, 0.45, 0.75, 0.90 and 1.05 mg/ml in the mobile phase and injected in triplicate. Linear regression analysis was carried out on the standard curve generated by plotting the concentration of 2-phenoxyethanol versus peak area response. The accuracy of the method was evaluated by performing recovery studies at three different levels (50, 100 and 140%) addition of 2-phenoxyethanol. Recovery and average recovery was calculated. The repeatability of the method was evaluated by assaying six replicate injections of the 2-phenoxyethanol at 100% of test concentration (0.75 mg/ml) and was expressed as relative standard deviation (RSD). Intermediate precision was demonstrated by two analysts using two LC systems and evaluating the relative peak area percent data across the two LC systems at three concentration levels (60, 100 and 120%) and expressed as RSD. The limit of detection (LOD) and limit of quantitation (LOQ) were evaluated by applying different dilutions of the standard solution of and peak area of analyte was plotted against concentration and calculated using formula: LOD = (3.3 σ)/s and LOQ = (10 σ)/s. The specificity of the method was confirmed by injecting extracted placebo sample to demonstrate the absence of interference with the elution of the 2-phenoxyethanol. Forced degradation studies were also performed to evaluate the specificity of 2-phenoxyethanol under four stress conditions (heat, UV light, acid, base).

## RESULTS AND DISCUSSION

Efficient chromatography and high sensitivity was achieved by using acetonitrile-tetrahydrofuran-water as the mobile phase with varying detection wavelengths, based on the response of the analyte. However, the analyte peak tailed badly on some C_18_ columns with this mobile phase with longer analytical run time. Using the C_8_ column minimised the tailing and shortened the run time. The amount of organic modifier was adjusted so that the assay run time could be reduced for faster analysis of samples. Chromatograms illustrating the separation of 2-phenoxyethanol reference standard and lubricant sample formulation are displayed in figs. 2a and 2b, respectively confirming specificity with respect to 2-phenoxyethanol. The remaining chromatographic conditions listed in Section LC instrumentation and conditions were chosen for the following reasons: the lower flow rate of 1 ml/min was chosen because of the potential problems associated with elevated back pressures. The PDA UV detector was set at 258 nm, λ_max_ for 2-phenoxyethanol. Column temperature was held at 35° although separation at 30° and 40° indicated that slight variation in temperature did not have a significant effect on retention or peak shape. Therefore, results produced at 35° were more reproducible and stable compared to those produced at 30° and 40°, and therefore 35° was selected as the working temperature. The injection volume of 10 μl and sample concentration of 0.75 mg 2-phenoxyethanol/ml in mobile phase were chosen to simplify sample preparation (further dilution is not needed). This concentration allows purity evaluation. The peak for 2-phenoxyethanol is well resolved within the linear range for UV detection. System suitability testing was performed by injecting ten replicate injections of a solution containing 0.75 mg 2-phenoxyethanol/ml. The percent relative standard deviation (RSD) of the peak area responses was measured, giving an average of 0.18 (*n* = 10). The tailing factor (T) for each 2-phenoxyethanol peak was 1.257, the theoretical plate number (N) was 4803, and the retention time (t_R_) variation %RSD was < 1% for ten injections. The RPLC method met these requirements within the accepted limits[9,13]. For the determination of method robustness within a laboratory during method development a number of chromatographic parameters were determined, which included flow rate, temperature, mobile phase composition, and columns from different lots, In all cases good separation of 2-phenoxyethanol were always achieved, indicating that the method remained selective for 2-phenoxyethanol preservative under the tested conditions. For stability study, samples and standard solutions were chromatographed immediately after preparation and then re-assyed after storage at room temperature for 48 h. The results in Table 1 show that there was no significant change (<1% response factor) in 2-phenoxyethanol concentration over this period.

**TABLE 1:** METHOD VALIDATION RESULTS OF 2-PHENOXYETHANOL

Validation step	Parameters	Concentration (mg/ml)	Results	Acceptance criteria
Standard stability	% change in response factor	0.75	0.08	X < 2
Sample stability	% change in response factor	0.75	0.10	X < 2
Linearity (*n* = 3; *k* = 5)	Correlation coefficient (r^2^)	0.15-1.05	y = 3238.7x- 380.33 (r^2^ = 0.9999)	r^2^ = ≥ 0.998
Repeatability (*n* = 10)	*t_R_* (min) (%RSD)	0.75	0.07	X < 1
	Peak area (%RSD)		0.11	X < 1
Intermediate precision (*n* = 6)	Instrument (%RSD)	0.75	0.22	X < 2
	Analyst (%RSD)	0.75	0.28	X < 2
System suitability	Peak area (%RSD)	0.75	0.08	X < 2

Linearity was studied using five solutions in the concentration range 0.15-1.05 mg/ml (20-140% of the theoretical concentration in the test preparation, *n*= 3). The regression equation was found by plotting the peak area (*y*) versus the 2-phenoxyethanol concentration (*x*) expressed in mg/ml. The correlation coefficient (r^2^= 0.9999) obtained for the regression line demonstrates that there is a strong linear relationship between peak area and concentration of 2-phenoxyethanol (Table 1). The analyte response is linear over the range of 80 to 120% of the target concentration for 2-phenoxyethanol assay.

The accuracy of the method was evaluated by means of recovery assay, adding known amounts of 2-phenoxyethanol reference standard to a known amount of lubricant formulation in order to obtain three different levels (50%, 100% and 140%) of addition. The samples were analysed and the mean recovery was calculated. The data presented in Table 2 shows the recovery of 2-phenoxyethanol in spiked samples met the evaluation criteria for accuracy (100 +/-2.0% over the range of 80 to 120% of target concentration).

**TABLE 2:** RECOVERY STUDIES OF 2-PHENOXYETHANOL FROM SAMPLES WITH KNOWN CONCENTRATION

Sample #	Percent of nominal	Amount of 2-phenoxyethanol (mg)		Recovery (%)*	RSD (%)*
		Added	Recovered		
1	60	3.012	3.013	100.03	0.56
2	60	3.014	3.012	99.93	
3	100	4.022	4.021	99.76	0.24
4	100	4.023	4.017	99.85	
5	140	5.019	5.002	99.66	0.17
6	140	5.022	5.018	99.92	
Mean				99.86	

* *n* = 3

Precision of the method was investigated with respect to repeatability (intra-day precision) and intermediate precision (inter-day variation). Repeatability of the method was evaluated by assaying six replicate injections of the 2-phenoxyethanol at 100% of test concentration (0.75 mg/ml). The %RSD of the retention time (min) and relative percent peak area were found to be less than 0.12% (Table 1). Intermediate precision (inter-day variation) was demonstrated by two analysts using two LC systems and evaluating the relative peak area percent data across the two LC systems at three concentration levels (60, 100 and 120%) that cover the assay method range (0.15-1.05 mg/ml). the mean and %RSD across the systems and analysts were calculated from the individual relative percent peak area mean values at the 50%, 100% and 125% of the test concentration. The %RSD values for both instruments and analysts were ≤ 0.28% (Table 1) and illustrated good precision of RPLC method.

**Fig. 3:**
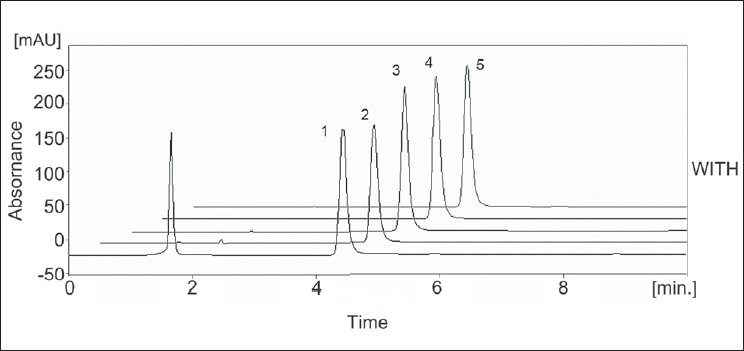
LC chromatograms of 2-phenoxyethanol under stress conditions. LC chromatograms of 2-phenoxyethanol under stress conditions: (1) acid degradation showing extra peak at retention time 1.65 min; (2) base degradation; (3) heat degradation at 60°; (4) fresh reference standard; (5) UV light degradation studies.

The RPLC-PDA/UV isoplot chromatogram demonstrates a good separation of the 2-phenoxyethanol. The isoplot chromatogram data consist of PDA UV/Vis absorption spectra from 200 to 300 nm for each point along the chromatogram. Injections of the extracted placebo were also performed to demonstrate the absence of interference with the elution of the 2-phenoxyethanol. These results demonstrate that there was no interference from the other materials in the lubricant formulation and, therefore confirm the specificity of the RPLC method. Forced degradation studies were performed to evaluate the specificity of 2-phenoxyethanol under four stress conditions (heat, UV light, acid, base). Solutions of 2-phenoxyethanol were exposed to 60° for 1 h, UV light using a UVL-56 lamp for 24 h, acid (1 M HCl) for 24 h and base (1 M NaOH) for 4 h. A summary of the stress results (retention time (t_R_), peak area, resolution (R) and theoretical plate numbers, (N) is shown in Table 3. Under acid (major degradation) and alkaline (minor degradation) hydrolysis conditions, the 2-phenoxyethanol content decreased and additional peaks were observed (fig. 3). No degradation was observed under other hydrolysis conditions (heat, UV light) studied. The addition peak detected at 1.65 min under acid and 1.95 min under alkaline conditions. This was further confirmed by peak purity analysis on a PDA UV detector. The 2-phenoxyethanol analyte obtained by acid hydrolysis was well resolved from the additional peak indicating the specificity of the method. In addition, the selectivity of the method was also checked by mixing all degradation samples and analysing by LC. No degradation peaks were found near to the 2-phenoxyethanol peak. As mentioned above, degradation was occur only under acidic conditions and resolution between 2-phenoxyethanol and degradation peak were >2 in each case.

**TABLE 3:** FORCE DEGRADATION STUDIES DATA OF 2-PHENOXYETHANOL

Stress conditions	Sample treatment	t_R_* (min)	Area (mAU s)	R*	N*	Degradation
Reference standard	Fresh solution	4.417	1854.90	-	4803	No degradation
Heat degradation	50° for 1 h	4.417	1754.25	-	6079	No degradation
Light degradation	UV Light for 24 h	4.417	1808.36	-	4803	No degradation
Acid degradation	1M HCl for 24 h	4.433	1590.07	16.42	6125	
		1.650	762.36	-	3394	Degradation
Base degradation	1M NaOH for 4 h	4.433	1538.41	2.64	6125	
		1.950	66.15	-	3033	Degradation

* t_R_: retention time; R: resolution; N: theoretical plate numbers

The LOD and LOQ of 2-phenoxyethanol was determined based on standard deviation (σ) of response and slope (s). 2-Phenoxyethanol solutions were prepared in the range 0.05-250 μg/ml and injected in triplicate. Average peak area of analyte was plotted against concentration. LOD and LOQ were calculated by using the following equations: LOD = (3.3 σ)/s and LOQ = (10 σ)/s. The LOD was determined to be 0.095 mg/ml and LOQ was found to be 0.15 mg/ml for 2-phenoxyethanol with %RSD less than 0.14% for six replicate injections.

A system suitability test was performed to determine the accuracy and precision of the system by injecting six replicate injections of 2-phenoxyethanol standard solution. The RSD of the peak areas responses was measured. The RSD for 2-phenoxyethanol was 0.08% as can be seen in Table 1.

A simple and rapid reversed-phase liquid chromatographic method with UV spectrophotometer detection was developed for the determination of 2-phenoxyethanol in senselle lubricant formulation. The method was validated and the results obtained were accurate and precise with RSD< 1 % in all cases and no significant interfering peaks were detected. The method is specific, selective, robust and reliable for routine use in quality control for analysis of 2-phenoxyethanol in bulk senselle lubricant samples, raw materials, and final products release.
